# Antineutrophil Cytoplasmic Antibodies-Associated Glomerulonephritis in Diabetic Kidney Disease

**DOI:** 10.7759/cureus.79955

**Published:** 2025-03-03

**Authors:** Germán A Landeros, Lauro F Amador, Miguel A Flores, Ma. D Sánchez, José O Juárez, Severo M Abraham, Omar A Jiménez, Jhoana D Vázquez

**Affiliations:** 1 Nephrology, Instituto Mexicano del Seguro Social, León, MEX; 2 Hematology, Instituto Mexicano del Seguro Social, León, MEX; 3 Pathology, Hospital Aranda de la Parra, León, MEX; 4 Forensic Medicine, Fiscalía General del Estado de Guanajuato, León, MEX

**Keywords:** adult nephrotic syndrome, anca-associated vasculitis, diabetic nephropathies, image-guided biopsy, pauci-immune crescentic glomerulonephritis

## Abstract

Diabetic kidney disease is a very prevalent complication in the context of type 2 diabetes. However, there is evidence showing a high variability in diagnosis when a kidney biopsy is performed. We present a case of a woman with a diagnosis of diabetic kidney disease, systemic arterial hypertension, obesity, and a high risk of progression of chronic kidney disease who presented with a sudden onset nephrotic syndrome and rapidly progressive deterioration of renal function. Kidney biopsy revealed pauci-immune extracapillary glomerulonephritis with acute thrombotic microangiopathy and class IIa diabetic nephropathy. Antineutrophil cytoplasmic antibodies (ANCA) and low complement were detected. The patient received treatment based on plasma exchanges, steroids with methylprednisolone and prednisone and intravenous cyclophosphamide with improvement of renal function. In conclusion, expansion of kidney biopsy criteria in patients with a diagnosis of type 2 diabetes is mandatory to provide adequate treatment and prognosis in the context of a high prevalence of alternative or concomitant disease.

## Introduction

Chronic kidney disease is the most relevant complication of type 2 diabetes. This entity, known as diabetic kidney disease is defined as: the presence of any impairment of renal function, albuminuria, or both of them in the context of diabetes. Half of people with type 2 diabetes and a third with type 1 diabetes will have these complications [1,2]. Worldwide, it is estimated that approximately 700 million people have diabetic kidney disease with a higher prevalence in women. There are risk groups such as African Americans, American natives, and Hispanics [2,3]. Diabetic kidney disease is the consequence of multiple processes. These alterations will lead to hyperfiltration, hypertrophy, and glomerular hypertension. The end of the natural history of the disease is, as in many etiologies of chronic kidney disease, glomerulosclerosis, and interstitial fibrosis [1,3].

Diagnosis is made by pathological anatomy and four stages have been described in type 1 diabetes, the most frequent changes associated with diabetic nephropathy are: glomerular basement membrane thickening, mesangial expansion with or without nodular sclerosis, podocyte effacement, and endothelial injury [2,4]. A workup based on clinical evaluation and physical examination, including laboratory and imaging studies, should be performed to determine the need for renal biopsy whenever there is suspicion of an entity other than diabetes that justifies diabetic kidney disease [3]. A meta-analysis of 48 studies showed that the majority of nephrologists do not promote kidney biopsy in patients with diabetes. The prevalence of an alternative diagnosis to diabetic nephropathy is higher in patients with diabetes, being more than 82% of all diagnoses. Of these, IgA nephropathy was the most common finding; other frequent findings were focal and segmental glomerulosclerosis and interstitial nephritis. However, only one study showed the presence of renal vasculitis with a prevalence of only 15% [5].

We present a case of ANCA-mediated vasculitis in a patient with type 2 diabetes of more than 15 years of evolution. This article was previously presented as a meeting abstract at the 2024 World Congress of Nephrology on April 16, 2024.

## Case presentation

Medical history

A 70-year-old woman with a family history of type 2 diabetes, systemic arterial hypertension, and ischemic heart disease. She was diagnosed with type 2 diabetes 15 years ago which has been treated with metformin 850 mg per day, NPH (neutral protamine Hagedorn) insulin 70 units subcutaneously per day, and rapid-acting insulin 15 units subcutaneously twice a day. Systemic arterial hypertension of 20 years of diagnosis on treatment with telmisartan 40 mg twice a day, spironolactone 25 mg twice a day. Grade 3 obesity with a BMI of 48.8 kg/m^2^. She consulted the nephrology department and reported a 3-month history of lower limb edema progressing to anasarca. Physical examination revealed a cervical circumference of 55 cm and abundant subcutaneous tissue. Initial serologic studies showed a serum creatinine of 2.9 mg/dl (estimated glomerular filtration rate at 16 ml/min/1.73 m^2^).

Additional investigation

A 24-hour urine collection and serologic studies were requested (Table 1).

**Table 1:** Laboratory findings ESR: erythrocyte sedimentation rate; P-ANCA: perinuclear anti-neutrophil cytoplasmic antibodies; HIV: human immunodeficiency virus; HBV: hepatitis b virus; HCV: hepatitis c virus

Laboratory data	Patient’s results	Reference
Creatinine (mg/dl)	2.9	0.7 – 1.5 (mg/dl)
Blood urea nitrogen (mg/dl)	76	7 – 20 (mg/dl)
Sodium (mEq/l)	135	137 – 145 (mEq/l)
Potassium (mEq/l)	3.9	3.6 – 5.0 (mEq/l)
Chlorine (mEq/l)	97	98 – 107 (mEq/l)
Bicarbonate (mEq/l)	20	22 – 26 (mEq/l)
Triglycerides (mg/dl)	373	<150 (mg/dl)
Total cholesterol (mg/dl)	170	35 – 200 (mg/dl)
C3 (mg/dl)	60	88 – 165 (mg/dl)
C4 (mg/dl)	9.0	14 – 44 (mg/dl)
ESR (mm/h)	29	0 – 10 (mm/h)
Anti-nuclear antibodies	1:160	-
P-ANCA (UR/ml)	190.8	< 20 (UR/ml)
HIV	Non-reactive	Non-reactive
HBV	Non-reactive	Non-reactive
HCV	Non-reactive	Non-reactive
Urinalysis		
Specific gravity	1.015	-
pH	5.0	-
Protein (mg/dl)	100	0 (mg/dl)
Leukocytes (per field)	10 - 15	-
Erythrocytes (per field)	25 - 30	100% erythrocyte dysmorphism
24 – hour urine collection		
Volume (ml)	2000	-
Protein quantification (mg)	5700	-

Diagnostic approach

**Figure 1:**
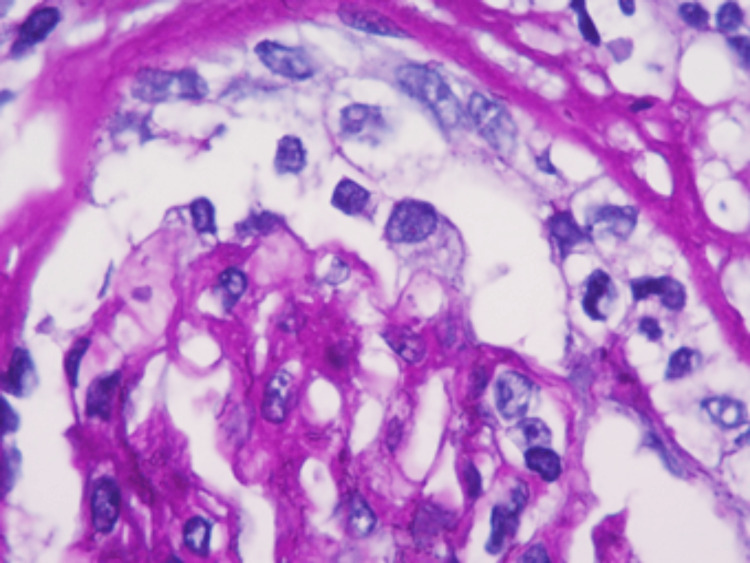
Active glomerular lesion. PAS (Periodic acid Schiff) staining

**Figure 2:**
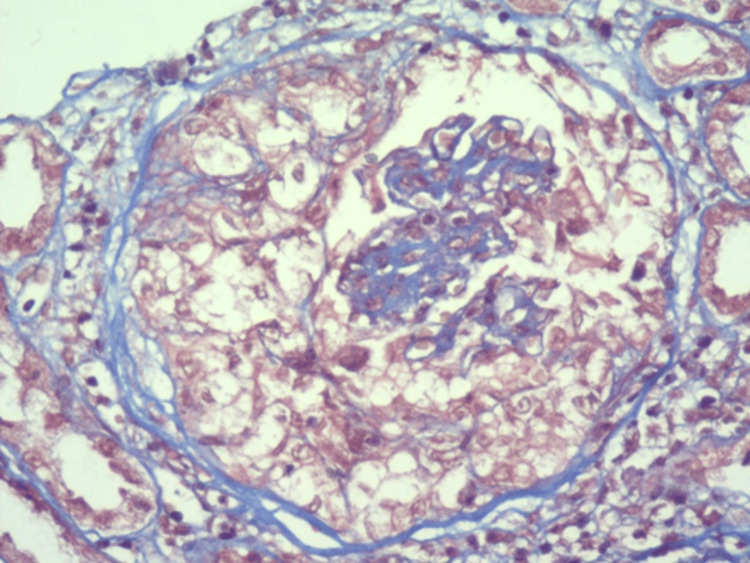
Active glomerular lesion. Masson trichome staining

**Figure 3:**
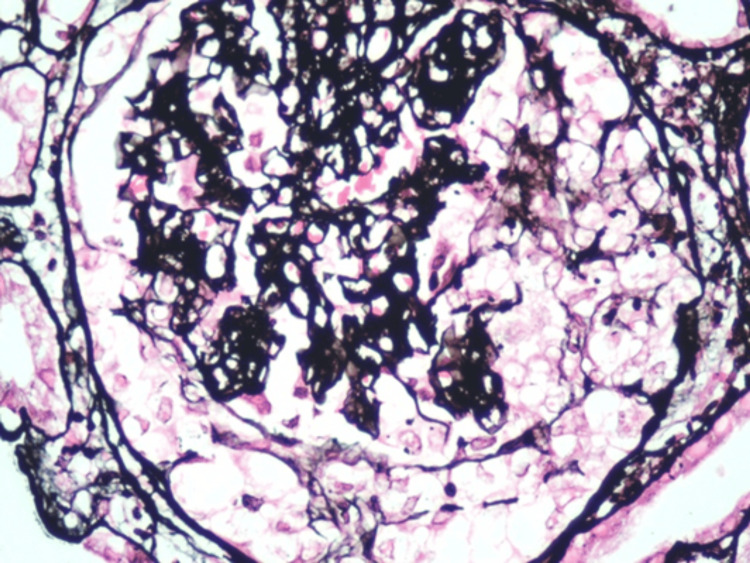
Active glomerular lesion. Jones Methenamine Silver staining

**Figure 4:**
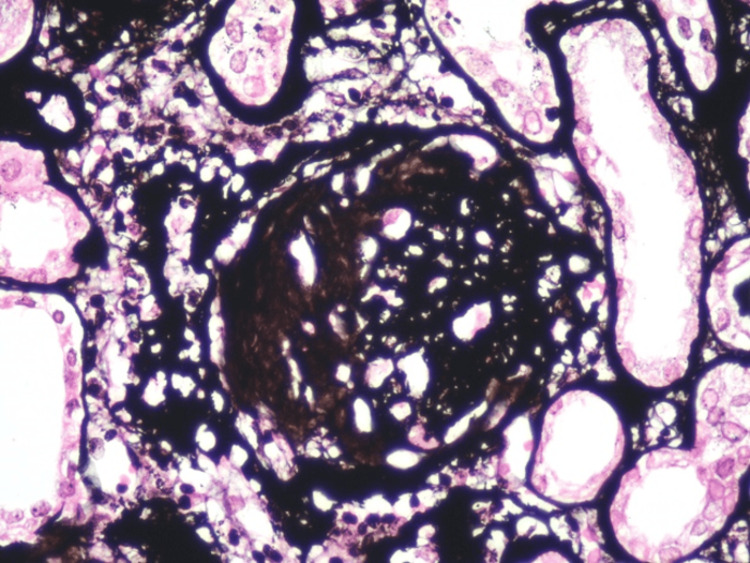
Chronic glomerular lesion. Jones Methenamine Silver staining

**Figure 5:**
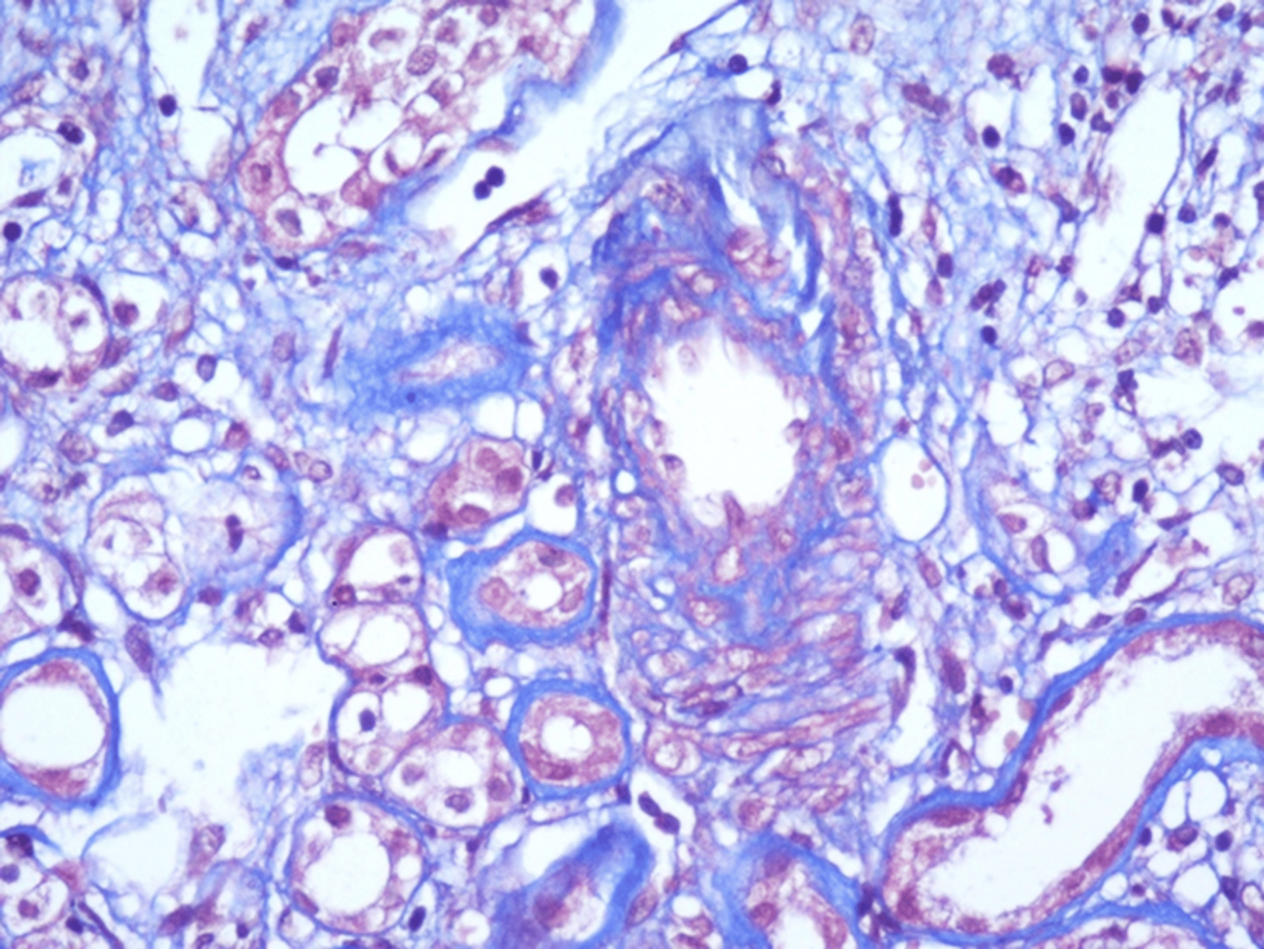
Vascular lesion (Acute thrombotic microangiopathy). Masson trichrome staining

**Figure 6:**
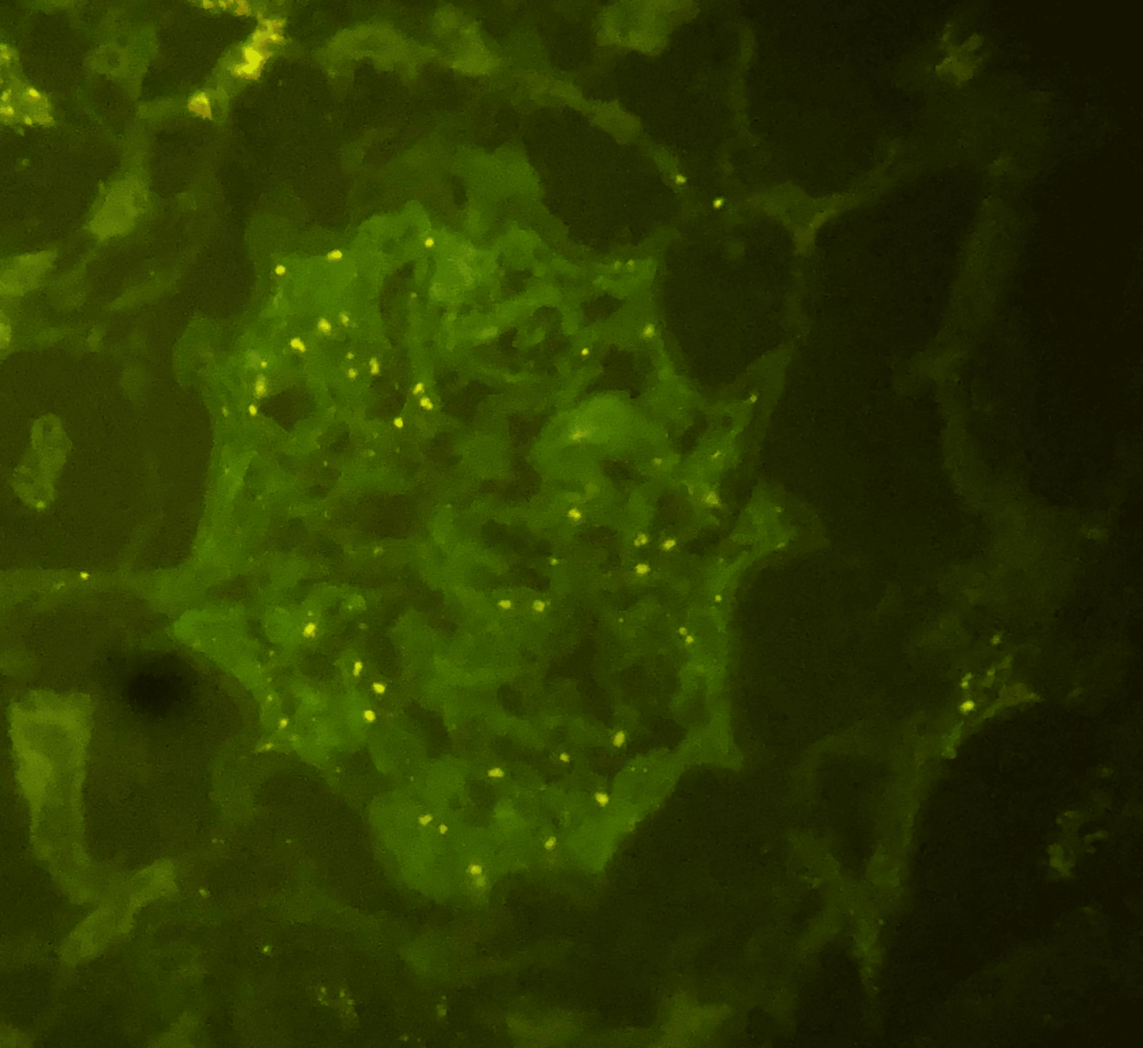
Immunofluorescence stain. Non-reactive.

It was decided to hospitalize the patient to perform a kidney biopsy in which two passes were performed, obtaining two cylinders with kidney tissue for microscopic evaluation. A diagnosis of pauci-immune extracapillary glomerulonephritis with acute thrombotic microangiopathy, class IIa diabetic nephropathy (The Renal Pathology Society (RPS)), moderate interstitial fibrosis (30%) and moderate tubular atrophy (30%) was made (Figures 1-6).

Follow-up care

The patient was admitted to the hospital to start treatment at this point with an elevation of creatinine to 4.5 mg/dl. Blood urea nitrogen 55 mg/dl. Electrolytes were normal. Treatment was initiated based on plasma exchanges with albumin, a total of four sessions during hospitalization, combined with boluses of methylprednisolone at a dose of 500 mg for three doses with subsequent dose reduction of steroid with prednisone at a dose of 1 mg per kg. Cyclophosphamide at a dose of 8 mg. per kilogram of ideal body weight for six doses with 2 weeks between each dose for the first three doses and three weeks between each dose for the last three doses. Follow-up was provided 3 weeks after completing treatment demonstrating improvement in renal function with a creatinine of 1.0 mg/dl with an estimated glomerular filtration rate of 53.7 ml/min/1.73 m^2^ of body surface area by CKD EPI. A chest CT scan was performed without evidence of pulmonary lesions.

## Discussion

The relevance of performing a renal biopsy in a patient with type 2 diabetes is to provide specific treatment and to identify those at risk of recurrence in case of renal transplantation [6]. Indications for renal biopsy in a patient with type 2 diabetes are: isolated microscopic hematuria, sudden or progressive onset nephrotic syndrome, rapidly progressive deterioration of renal function, and chronic kidney disease without proteinuria. However, it is worth commenting on three other well-founded indications: to establish a prognosis of diabetic nephropathy, to make the diagnosis of classic diabetic nephropathy without proteinuria, and to identify an alternative entity to establish recommendations related to future renal transplantation [7].

In a retrospective study of 611 renal biopsies in patients with type 2 diabetes, 37% showed isolated diabetic nephropathy, 36% non-diabetic renal disease with focal and segmental glomerulosclerosis as the most frequent diagnosis, and pauci-immune glomerulonephritis the most infrequent. Twenty-seven percent showed diabetic nephropathy with non-diabetic renal disease, in this group acute tubular necrosis was the most frequent diagnosis. In this study, the duration of diabetes was the most relevant factor for ruling out non-diabetic kidney disease [8].

In another retrospective study where 832 renal biopsies were performed in patients with type 2 diabetes, 39.5% had isolated diabetic nephropathy, 49.6% had non-diabetic renal disease and 10.8% had a combination of the above. The most prevalent diagnoses were in descending order: nephroangiosclerosis, IgA nephropathy, and membranous nephropathy. Again, extracapillary glomerulonephritis was the most infrequent [9].

These findings suggest the relevance of performing a renal biopsy in a patient with type 2 diabetes and chronic kidney disease. The present case is interesting because the patient has multiple risk factors for metabolic syndrome, long-standing diabetes, and rapidly progressive renal function deterioration with pauci-immune glomerulonephritis.

The presentation of pauci-immune glomerulonephritis is most common in patients with serological evidence of ANCA autoantibodies, and to date, we have identified only 10 patients with ANCA-associated glomerulonephritis and type 2 diabetes [10,11]. The prevalence of anti-MPO antibodies in type 1 diabetes has been identified as 38% but is infrequent in type 2 diabetes [12].

## Conclusions

The presentation of this clinical case is important because it highlights the importance of timely identification of alternative nephrological diagnoses to the main diagnostic suspicion. In this case, the presentation of a patient with long-standing type 2 diabetes associated with morbid obesity and systemic arterial hypertension without adequate control makes diabetic nephropathy one of the possible diagnoses. However, the presence of atypical features in the clinical presentation should set the tone for suggesting a percutaneous renal biopsy in order to objectively establish a diagnosis that may even be subject to a treatment that modifies the natural course of the disease. Therefore, we suggest expanding the indications for renal biopsy in patients with long-standing type 2 diabetes.
